# “I just keep thinking that I don’t want to rely on people.” a qualitative study of how people living with dementia achieve and maintain independence at home: stakeholder perspectives

**DOI:** 10.1186/s12877-019-1406-6

**Published:** 2020-01-03

**Authors:** Penny Rapaport, Alexandra Burton, Monica Leverton, Ruminda Herat-Gunaratne, Jules Beresford-Dent, Kathryn Lord, Murna Downs, Sue Boex, Rossana Horsley, Clarissa Giebel, Claudia Cooper

**Affiliations:** 1 University College London, London, UK; 2 UCL Division of Psychiatry, 6th Floor Wing A, Maple House, 149 Tottenham Court Road, London, W1T 7NF UK; 3 University of Bradford, Bradford, UK; 4 Alzheimer’s Society, London, UK; 5 NIHR ARC NWC and Institute of Population Health Sciences, University of Liverpool, Liverpool, UK

**Keywords:** Qualitative, Dementia, Independence, Psychosocial intervention, Support systems, Functioning

## Abstract

**Background:**

Most people living with dementia want to remain in their own homes, supported by family and paid carers. Care at home often breaks down, necessitating transition to a care home and existing interventions are limited**.** To inform the development of psychosocial interventions to enable people with dementia to live well for longer at home, we qualitatively explored the views of people living with dementia, family carers and health and social care professionals, on how to achieve and maintain independence at home and what impedes this.

**Methods:**

We conducted an inductive thematic analysis of qualitative interviews with 11 people living with dementia, 19 professionals and 22 family carers in England.

**Results:**

We identified four overarching themes: being in a safe and familiar environment, enabling not disabling care, maintaining relationships and community connectedness, and getting the right support. For people living with dementia, the realities of staying active were complex: there was a tension between accepting support that enabled independence and a feeling that in doing so they were accepting dependency. Their and professionals’ accounts prioritised autonomy and ‘living well with dementia’, while family carers prioritised avoiding harm. Professionals promoted positive risk-taking and facilitating independence, whereas family carers often felt they were left holding this risk.

**Discussion:**

Psychosocial interventions must accommodate tensions between positive risk-taking and avoiding harm, facilitating autonomy and providing support. They should be adaptive and collaborative, combining self-management with flexible support. Compassionate implementation of rights-based dementia care must consider the emotional burden for family carers of supporting someone to live positively with risk.

## Introduction

Dementia is a major global health challenge, with the number of people living with dementia worldwide set to triple from over 46 million to 131.5 million by 2050 [1]. Dementia profoundly impacts upon the person living with dementia, their family and carers and wider society, with the annual global cost estimated at 818 billion US dollars [1]. Most people diagnosed with dementia wish to continue living in their own homes as independently as possible [2–4], but with progressive functional dependence [5], this can be difficult.

There is a drive towards person-centred care in the community with policy initiatives to promote the functional capabilities and independence of people living with dementia [6]. These focus on valuing the person, upholding personhood, meeting psychological needs, adopting the person’s perspective and ensuring a supportive social environment for people living with dementia [7, 8] with aspiration towards “dementia-friendly community” “dementia positivity” and “dementia-capable alliance” [9]. Existing approaches to facilitating independence draw upon relationship-centred [10], rights-based [11], recovery [12], environmental [9, 13] and family carer based models [14] to inform understandings of how best to achieve this. However to date, only two psychosocial interventions have resulted in an increase in the time people with dementia remain living in their own homes [15–18].

Older people’s retention of independence has been conceptualised in policy and professional discourses as synonymous with staying in one’s own home for as long as possible. Older people themselves present a more nuanced perspective of independence; as accepting support, being able to do things alone, having informal support and financial resources and preserved mental and physical capacities [19]. For people living with dementia, there can be a tension between independence as an expression of autonomy [20, 21] and interdependence enabling people with dementia to remain in their own homes and communities [13]. Qualitative research has explored the ethical dilemmas faced by family carers and professionals in promoting the dignity and autonomy of people living with dementia [21, 22] and deciding how best to adapt the home environment to facilitate independence [23]. Less is known about how people living with dementia, family carer and professional perspectives differ.

Recent qualitative studies have privileged the voices of people living with dementia [24], exploring the impact of dementia upon quality of life [25] and dignity [26]. None have directly considered how people living with dementia in their own homes feel they can best achieve independence. Deeper understanding of the complex dilemmas inherent in providing person-centred care to people living with dementia in their own homes requires their voices to be heard and could be instrumental in increasing efficacy and acceptability of interventions promoting independence at home for people living with dementia.

This is the first qualitative study to explore how people living with dementia, family carers and health and social care professionals understand independence at home for people living with dementia. We consider how these stakeholders differ in their views of how to achieve and maintain independence at home and what impedes this.

## Methods

London (Camden and Kings Cross) National Research Ethics Service (NRES) approved the study (reference: 17/LO/1713) in November 2017.

### Setting and participants

We recruited family carers and people living with dementia via three UK National Health Service (NHS) memory services, a private home care service, an Alzheimer’s Society Experts by Experience group and the social media platform Twitter. Professionals were recruited via participating NHS memory services, University College London (UCL) and social services. We purposively selected participants to ensure we interviewed people of both genders and differing ages, ethnicities, nationalities, roles (professionals), relationships to the person living with dementia (family carers) and experiences. We recruited in urban, semi-rural and rural areas. Where possible we interviewed dyads of people living with dementia and their relative either together or separately (depending on their preferences), but overall, because we were recruiting from a range of services, not everyone had a family carer available and others declined to take part, only a proportion of our sample were matched.

### Procedures

All participants gave written informed consent (we did not recruit people living with dementia unable to give informed consent - trained researchers assessed this). AB, ML, JBD, PR and RHG conducted interviews in participants’ homes, work places or University offices. Interviews followed a semi-structured interview schedule (see Additional file 1) based on the literature, and research team expertise, with slightly amended versions for people living with dementia, family carers and professionals.

The research team met weekly to review recruitment and reflect on initial themes. We ceased interviews after reaching thematic saturation, the point at which no further themes emerged from discussions and reflections on additional interviews [27]. Interviews were audio recorded, transcribed verbatim and anonymised.

### Data analysis

We used NVivo 11 software, taking an inductive thematic analytic approach [28]. Two co-authors independently and systematically coded each transcript into meaningful fragments and labelled these initial codes. We met to resolve discrepancies and discuss initial themes corresponding to our research questions. PR and AB then organised the data into a coding frame, using the constant comparison method, to identify similarities and differences in the data [29]. We presented and discussed an initial summary of the thematic analysis to an advisory group of family carers, professionals and academics involved in our project using their feedback to revise our analysis, to enhance the credibility of our findings.

We then revisited the codes looking at commonalities and differences between the person living with dementia, family carer and professional accounts, integrating this into our thematic analysis. We present a checklist of methods used against the Standards for Reporting Qualitative Research in Additional file 2 [30].

## Results

### Study participants

Between April and August 2018 we interviewed 11 people living with dementia (PLWD), 22 family carers (FC) and 19 health and social care professionals (HSCP). We recruited participants from memory services in London (*n* = 20), South West (*n* = 10) and Northern (*n* = 10) England. We also recruited two family carers from a London care agency and two through Twitter. We recruited one family carer and one person with dementia from Experts by Experience groups. Two professionals were recruited through links with UCL, three from North London social services and one through South West England social services.

Characteristics of participants are presented in Tables 1, 2 and 3. Of the people living with dementia that were interviewed, five were interviewed alone and not matched (i.e. their carer did not take part in an interview), three had a carer present in their interview but the carer did not take part in an separate interview, two had a carer present in their interview and their carer also took part in a separate interview, and one did not have their carer present but the carer took part in a separate interview.

**Table 1:** Characteristics of people living with dementia

Characteristics		*n* (%) or mean (*SD*)
Age		78.6 (7.8)
Gender	Female	5 (45.5)
	Male	6 (54.5)
Ethnicity	White British	8 (72.2)
	White other Other - Asian	1 (9.1) 1 (9.1)
	Other Mauritian	1 (9.1)
Marital status	Married/with partner	4 (36.7)
	Single	3 (27.3)
	Divorced	1 (9.1)
	Widowed	3 (27.3)
Living arrangements	Lives alone	5 (45.5)
	Lives with relatives	6 (54.5)
Type of dementia	Alzheimer’s disease	3 (27.3)
	Vascular	2 (18.2)
	Other	2 (18.2)
	Not specified	4 (36.7)
Time since diagnosis	1–3 years	4 (36.7)
	3–5 years	2 (18.2)
	5–10 years	1 (9.1)
	Not specified	4 (36.7)

**Table 2:** Characteristics of family carers

Characteristics		*n* (%) or mean (*SD*)
Age		57.7 (14.3)
Gender	Female	12 (54.5)
	Male	10 (45.5)
Ethnicity	White British	9 (40.9)
	Indian	6 (27.3)
	Bangladeshi	4 (18.2)
	Other	3 (13.6)
Marital status	Married/with partner	13 (59.1)
	Single	5 (22.7)
	Divorced	3 (13.6)
	Widowed	1 (4.5)
Employment	Retired	9 (40.9)
	Part time	5 (22.7)
	Unemployed	3 (13.6)
	Other	3 (13.6)
	Full time	2 (9.1)
Relationship to person with dementia	Son/daughter	11 (50)
	Husband/wife/partner	6 (27.3)
	Niece	2 (9.1)
	Friend	1 (4.5)
	Sibling	1 (4.5)
	Daughter-in law	1 (4.5)
Living with person with dementia	Yes	15 (68.2)
	No	7 (31.8)
Type of dementia	Alzheimer’s disease	8 (36.4)
	Vascular	4 (18.2)
	Other	4 (18.2)
	Not specified	6 (27.3)
Length of time caring for relative	1–3 years	8 (36.4)
	3–5 years	2 (9.1)
	5–10 years	2 (9.1)
	10+ years	5 (22.7)
	Not specified	5 (22.7)

**Table 3:** Characteristics of health and social care professionals

Characteristics		*n* (%) or mean (*SD*)
Age		41.4 (10.9)
Gender	Female	13 (68.4)
	Male	6 (31.6)
Ethnicity	White British	9 (47.4)
	White other	5 (26.3)
	Indian	2 (10.5)
	Other	3 (15.8)
Professional role	Commissioner	3 (15.8)
	Social worker	2 (10.5)
	Dementia lead	2 (10.5)
	Service manager	2 (10.5)
	Psychologist	2 (10.5)
	Support worker	2 (10.5)
	GP	2 (10.5)
	Geriatrician	1 (5.3)
	Nurse	1 (5.3)
	Physiotherapist	1 (5.3)
	Psychiatrist	1 (5.3)
Time working in dementia care	1–3 years	1 (5.3)
	3–5 years	5 (26.3)
	5–10 years	5 (26.3)
	10+ years	8 (42.1)
Personal experience of dementia	Yes	12 (63.2)
	No	7 (36.8)

### Qualitative findings

We identified four themes common across participants living with dementia, family carers and professionals. These were: being in a safe and familiar environment, enabling not disabling care, maintaining relationships and community connectedness, and getting the right support. Each of the overarching themes incorporate all three stakeholder group perspectives and represent a shared understanding of what independence at home ‘should’ include. The sub themes presented include factors that facilitate or impede this being achieved and how it impacts on each stakeholder group (see Table 4). We discuss these below, highlighting key tensions and where stakeholders’ perspectives differed.

**Table 4:** Table of themes and differences between stakeholder groups

Overarching theme	Sub theme	Stakeholder contribution			Notable differences and tensions between stakeholder groups
		PLWD	FC	HSCP	
1.1. Being in a safe and familiar environment	1.1 Adapting the environment	x	x	x	Family carers focus on reducing the risk of potential harm within the home vs PLWD and professionals promoting adaptations to enhance functioning at home and outside. Family carers want to support relatives to remain independent but fear negative consequences and may act to protect or reduce potential risk by imposing constraints. This negatively impacts on the independence of PLWD.
	1.2 Balancing autonomy and minimising harm	x	x	x	
1.1.2. Enabling not disabling care	2.1 Building on preserved abilities		x	x	All stakeholder groups described the importance of an ‘enabling’ approach and PLWD living well. As they would like, at home. Professionals have a clear idea of why and how this can be done however for family carers, this can be difficult to enact and requires them to tread a fine line and with a potentially disabling consequence for the PLWD.
	2.2 Supporting without undermining		x	x	
	2.3 Families want to protect and care	x	x	x	
1.1.3. Maintaining relationships and community connectedness	3.1 Making a contribution vs not being a burden	x	x	x	Having varied and positive relationships both within and outside of the family and home was promoted across groups. PLWD described the impact of dementia and the stigma connected to the disease as barriers to staying connected and feeling useful, whereas families described the complexities both practically and emotionally of supporting relatives to stay connected. The impact of dementia itself and the consequence of having to do more for relatives left family members and PLWD describing negative changes in their relationships and roles. Professionals acknowledged and could account for these changes but not what to do about them.
	3.2 Getting out and about	x	x	x	
	3.3 Changing roles and relationships	x	x	x	
1.1.4. Getting the right support	4.1 Difficulties in accessing services		x		All stakeholders felt that formal and informal support network were important but for family members, they were often caught between trying to navigate often inaccessible services and their relative’s reluctance to accept additional support. PLWD described how accepting additional support could undermine their independence and they worried about burden on their relatives. Professionals, although recognising their role in addressing different needs of carers and PLWD, frequently described trying to educate or persuade the carer about how best to respond.
	4.2 Balancing different needs		x	x	
	4.3 Reluctance to accept support	x	x	x	

*PLWD* People living with dementia, *FC* Family carers, *HSCP* Health and social care professionals

#### Being in a safe and familiar environment

Stakeholders agreed that this was integral to maintaining independence at home. People with dementia spoke about finding it easier to get around and function safely in their own home. Family carers considered that familiarity offered their relatives emotional comfort and security as it linked to past relationships and personal history.

The familiarity of being here of having her own things about her. She's always loved her bedroom and loved having her little possessions around her and pictures and her art work (FC-Sibling, lives with relative)

Professionals’ narratives focussed on avoiding the potential problems triggered by unfamiliar environments.

It's horrible to be taken into an alien environment, I think there's a real tension a lot of the time around how that works. Recognising, the value of people being in familiar environments, with the destructive nature of unfamiliar environments. (HSCP-Commissioner1)

##### Adapting the environment

People living with dementia and professionals described how specific adaptations to the physical environment facilitated or enhanced functional independence.

And then I caught on, I’ve got to have everything eye level. I can’t look down on anything. And because that was on the table and I’m looking down on it, I couldn’t work it… I’ve got to set it now on top of the toaster. (PLWD-Male1, lives alone)

We recently painted the toilet doors bright yellow so that people can find them and don’t get lost. They can go to the toilet on their own and that is a massive difference. (HSCP-Physiotherapist)

In contrast, family carers mostly discussed adaptations to the home environment designed to reduce risks of harm or increase safety, particularly when the person with dementia lived alone, which may ultimately reduce autonomy for the person living with dementia.

##### Balancing autonomy with minimising harm

Family carers’ accounts described tension between these two positions. Decisions to maintain autonomy could be distressing and onerous as they may (or had in the past) result in their relative coming to harm. Where safety was prioritised, decisions were often taken against their relative’s wishes.

Family members sometimes have to sit back and just let the other person make mistakes…That's a disparity, it's clear and it does cause tension, it does cause anxiety and I don't think one could get rid of that (FC-Niece, does not live with relative)

While for families, decisions were individual, personal and complex, professionals described rights-based approaches, and policy-based decision-making, documented and shared with families. They drew upon professional expertise to reinforce the need for risk to be tolerated and managed. In this way, their position was more aligned to that of people living with dementia but did not always acknowledge the complex position faced by family carers.

Because I think families just automatically see risk don’t they? But we can do managed risk. There’s lots of things we can put into place now. (HSCP-Physiotherapist)

People living with dementia lived with the consequences of this tension, where decisions may be taken by family which are counter to the person’s wishes or preferences.

If I'm alright and I feel good for myself I have to go. But they [family] will not let me go in case something happens outside. That’s the only reason that I can't go anywhere. (PLWD-Female1, lives alone)

#### Enabling not disabling care

Participants discussed the needs of people living with dementia for support to make choices and live as they wish. Professionals referred to ‘reablement’, ‘facilitative’ and ‘positive models’ and people living with dementia’s right to choice and autonomy.

If we promote facilitative models, there will be…Still be some people who become very disabled by their dementia…But there’ll be a much more significant proportion for whom it’s a condition that they are able to manage. (HSCP-Dementia lead)

Although there was agreement that this approach was key to promoting independence, for family carers there were challenges to enacting this approach and very real consequences for people living with dementia who described the disabling effect of other people doing activities for them.

Say the bloke has developed dementia, the wife will start doing it and, no, no you can’t do that…And she’ll go and do it, and then he’s sitting there. And it finishes up he’s going to forget because he’s not doing it. (PLWD-Male1, lives alone)

Although family carers shared this view, they struggled to put it into practice and perceived that professional advice often overlooked this reality.

The nurses have said you should get up every hour and move about but to get her motivated to do that, if I try and get her to do things, I'm nagging. (FC-Sibling, lives with relative)

##### Building on preserved abilities

Participants put this enabling approach into action by focusing upon the skills of the person living with dementia, highlighting the need to tailor responses to individual abilities. For health professionals this was an explicit approach including assessment of functioning and targeting of interventions and they identified positive effects of such an approach.

I took her to this church service and it was her leading me because this is what she knew very well. She was telling me stand up, sit down, hark, sing… And it was just a whole transformation because we were actually working with her skills. (HSCP-Manager)

Unsurprisingly, this was a more personal process for family carers who described adapting existing relationships and using knowledge of their relative to inform their approach.

##### Supporting without undermining

Family carers described maintaining a delicate balance in offering support, keeping in the background but staying close by, monitoring “subtly in the mirrors” (FC-Son1, lives with relative). This represented a rather precarious position which was difficult to sustain at times and required vigilance.

I don’t want to cramp her style. I let her have the independence. But when she comes I don’t make it obvious to her. I go inside and make sure everything is switched off, and if it’s not, I switch it off, but I don’t tell her about it, because you don’t want to embarrass somebody. (FC-Husband, lives with relative)

Professionals held a more detached position, talking more about giving people with dementia space to complete tasks, letting people know support was available if needed. This connected with being able to take a different position in relation to perceived risks, ultimately they were able to privilege their roles as facilitators rather than simply as protectors.

I think it’s essentially, especially if they’re on their own, but it’s essentially knowing that there’s some help and support there if needed. (HSCP-Commissioner2)

##### Families ‘do whatever it takes’ to protect and care

Families described doing “whatever it takes” (FC-Husband, lives with relative) to keep their relatives at home, often linking this to the unwanted alternative of moving to a care home; equating living at home and independence.

There's a time when you bully your family members into doing things, because you know that it's best for them. (FC-Niece, does not live with relative)

Professionals and people with dementia recognised this commitment but also worried it could have a paradoxically disabling effect, suggesting that in taking this protective position they inadvertently undermine independence.

It’s for the kindest of reasons, it really is, but it’s not; it’s the worst thing in the world that you [family] can do…they had trouble seeing that until I explained the consequence. (PLWD-Female2, lives alone)

Family members take over and I’m sure they do with the best will in the world. But because, you know, they’re worried and because they take over that person then loses their skills and they deteriorate more rapidly. (HSCP-Manager)

#### Maintaining relationships and community connectedness

Stakeholders considered this central to independence at home. Family carers spoke of the benefits of people with dementia having contact with immediate family, but less about wider relationships. When they did, supporting these interactions was often challenging but beneficial.

He had a phone call last night from his ex-neighbour. Before I gave him the phone I had to, and I did tell the neighbour, I’ve done it before, I said, I’ll just tell him. I had to explain who this was, where he was, and then you could see the, oh okay. It’s like a deck of cards falling into place. (FC-Son1, lives with relative)

Professionals suggested that, where possible, time away from family could support independence.

Yes, we kind of felt the best way for her to be independent would be through socialising maybe, a little bit away from the family. She was quite social once you gave her the time and allowed her, I guess, the space and the patience to be able to find the words. (HSCP-Psychologist)

Although some people with dementia spoke about engaging in social interaction as an outward sign of functioning, a way to show “dementia’s not beaten you” (PLWD-Male1, lives alone), they also described dementia symptoms and stigma surrounding the diagnosis as barriers to socialising. Some found interactions linked to another aspect of their identity, such as a hobby or occupation, easier.

I go around talking to people, vapers, and we’ll try and explain what liquids, what coils we’re building. The funny thing is that the majority of them [vapers] know I’ve got dementia, but they still treat me as an equal. They don’t frown on you, they don’t push you to the side. (PLWD-Male1, lives alone)

##### Making a contribution vs not being a burden

Many participants described how feeling valued, validated and having a purpose was integral to maintaining independence. Professionals considered supporting people to connect with aspects of their identity unrelated to dementia as integral to their role. People living with dementia equated trying to maintain independence and make a contribution with self-reliance and not needing support.

Because I can’t imagine anything more horrible than losing…that sense of being reliant on other people doing. (PLWD-Female2, lives alone)

When this was not possible, when they were not able to ‘support without undermining’ or protect their relatives from the impact of dementia, family members were conscious of the emotional impact upon their relatives and the distress this caused for themselves.

She goes, oh, I'm still the mother. You know, she doesn't want to come to us crying saying that. She'll get angry at us, saying, oh, this and that, but she doesn't want to show you that she's weak. (FC-Son2, lives with relative)

##### Getting out and about

Although perceived by all as in important aspect of ‘staying connected’, family carers spoke about the challenge of getting their relatives out of the house, especially if their relatives needed additional support or had complex health needs.

But my parents are so poorly, that they physically need help getting ready first of all, then holding their hands, getting them to that vehicle, they will get lost just from here to downstairs. (FC-Son3, lives with relative)

Professionals’ accounts did not reflect this complexity. Getting out was perceived as essential to an enabling approach. People living with dementia described how the stigma surrounding dementia influenced other peoples’ responses.

They always insist I get on the bus first. They always ring the bell for me coming home to make sure I’m getting off, but none of them will mention the word dementia. They won’t, because it’s that fear again, even though they see me and I have dementia; they won’t say the words. (PLWD-Female2, lives alone)

They also described fear resulting from dementia symptoms and the desire to overcome this to continue going out.

When I started realising what was happening with it, with the ground, with me, it was moving, I was actually, battle myself because I started to feel I’m not going out. And then I thought, well hold on, I’ve got it in my head if I don’t go out, dementia sets in. (PLWD-Male3, lives with family)

##### Changing roles and relationships

Family carers used evocative language - referring to being a “jailer” (FC-Sibling, lives with relative) - to describe perceived shifts in power and control in relationships with their relative. This was more common among children caring for parents.

I am trying not to be the dictator in the relationship. I do find myself, they always say you find yourself talking as your parents used to talk when you were a child, and I find that quite ironic. I am now “the parent”, in inverted commas, giving my father instruction. (FC-Son1, lives with relative)

People living with dementia also raised this in their accounts, aware of the changes in status and discussing the personal impact of this loss.

I used to be her boss. It’s changed. (PLWD-Male4, lives with family)

A number of professionals commented upon this changing dynamic, and sometimes linked it to abuse. Although they could account for and explain these difficulties, they tended to empathise with the position of the people living with dementia, and their need for protection, overlooking the family carers potential need for support.

So if the relationship hasn't been healthy, and the attachment's quite poor, from the adult to the child… The person, the parent has, say, dementia, can often flip the power dynamics that just doesn't…It's not right. And it can… It is abusive. (HSCP-Social worker)

#### Getting the right support

People living with dementia, family carers and health and social care professionals explained that independence at home was achieved and maintained via diverse and collaborative informal and formal support networks.

##### Difficulties in accessing services

All stakeholders discussed challenges accessing services impacting upon independence, but this was discussed most by family carers who described navigating and negotiating complex systems, sometimes not knowing where to start, or what was available.

In terms of picking up on the signs, advising family members, getting things moving. If I hadn’t pushed; if I hadn’t fought, you know, I made a real nuisance of myself, in order to get things going. (FC-Son4, lives with relative)

They described support as contingent on deterioration, rather than services promoting a more proactive, preventative or enabling approach.

It’s not like I want [PLWD] to deteriorate more so that I can say oh, now they qualify for these people and finally I can pay them. I don't want that. I don't want them to become worse (FC-Son3, lives with relative)

##### Balancing different needs

Health professionals felt part of their role was to balance the needs and goals of families and people living with dementia, explaining that when difficulties occurred it was often because of incompatible expectations.

I definitely think that it shouldn’t be just about the carer. Because sometimes I find that what the carer’s problem, issue, concern, whatever you wish to call it, isn’t necessarily bothering the person with dementia. (HSCP-Support worker1)

Often the professionals would privilege the perspective of the person with dementia, and seek to educate or change the perspective of family carers.

I say no leave him because to him he’s happy with that you know and he’s tried, he’s been independent doing that. Yes but…does it matter? Confrontation is just not worth it you know? (HSCP-Support worker2)

Family carers spoke extensively about the challenge of balancing their own needs against those of their relative when promoting independence.

So then I won’t be able to work, and I worry about my work. Will they sack me because I’m taking too many time off and I don’t come back to work? (FC-Wife, lives with relative)

##### Reluctance to accept support

Family carers described attempts to encourage relatives to accept support from outside the family, highlighting the personal impact. This may inadvertently reinforce the fear of being a burden described by people living with dementia but place them in a double bind since accepting more support may also further undermine independence.

You do need help mum. I say to her: I can't do this anymore, you know? I can only do what I can. (FC-Son5, lives with relative)

People with dementia spoke of their reluctance to accept care. Although support was intended to promote independence, the act of accepting support was itself a sign of dependence that they resisted.

The doctor was getting my tablets into blister pack, or whatever they call. I didn’t like it because this means that it’s so easy…I didn’t want it. I said to the GP, I want to use my brain and just give my tablet with a pack (PLWD-Male2, lives with family)

People living with dementia described how they would minimise demand on their family members by not asking for help, sometimes at personal cost.

If possible I don’t want them not to go to work because they have their own family. So I just wait or tell them that after they're finished they have to come here. (PWD-Female1, lives alone)

## Discussion

### Main findings

To our knowledge, this is the first qualitative study to consider how people living with dementia, family carers and professionals agree and differ in their understanding of how to achieve and maintain independence at home and what impedes it. We identified four overlapping themes: Being in a safe familiar environment, enabling not disabling care, maintaining relationships and community connectedness, and getting the right support. The process of achieving and maintaining independence is complex and requires not just adapting physical environment, but ongoing psychological and interpersonal adaptation (and negotiation) and a welcoming community.

Stakeholders agreed that a person-centred approach prioritising the inherent value of all people with dementia, seeking to uphold rights and promote abilities was important. Professionals often perceived such an approach as being relatively straightforward, while family carers and people living with dementia pointed to a myriad of ethical and practical dilemmas [21, 23]. For family carers these dilemmas often linked to the perceived safety of their relative and the family carer’s sense of personal responsibility. For people with dementia, the act of accepting support was paradoxically both an outward sign of dependence and an enabler of independence. Dichotomous notions such as doing things for oneself versus accepting support seem too simplistic to explain these contradictory positions. Concepts such as *relative interdependence*, *delegated autonomy* and *social and spatial independence* have previously been applied to understanding independence in older people living in a range of residential settings [19]. These concepts feel especially relevant to the nuanced and complex experiences of people living with dementia and those caring from them and the dynamic nature of interactions and negotiations around independence discussed in this paper.

### Clinical implications

These findings highlight the relational nature of independence at home: the dynamic interaction between the person living with dementia, the family carer and professional support, situated within a resource-limited, wider societal context [9, 10, 13]. Professionals were responding to ‘living well with dementia’ or ‘dementia positive’ agendas, perhaps underestimating the complexity and textured nature of the lived experiences of family carers and people with dementia themselves. Professionals promoted positive risk taking whereas family carers felt they were left holding this risk or persuading relatives to act in a particular way. For people living with dementia the realities of getting into the community and playing an active part were far from straightforward. Supportive interventions need to align these various tensions [31]. Interventions that have successfully increased time lived at home were multicomponent, incorporating a focus on communication and relationships, the needs and goals of both people living with dementia and their family members and linkage or referral to other resources [15–17].

We have built our findings into a multi-component intervention - New Interventions for Independence in Dementia (NIDUS family) that we are currently testing in England. The intervention is tailored around the goals of the person living with dementia, the needs of the family caregiver and takes an adaptive and collaborative approach, combining self-management [32] with flexible support, targeting change at individual, family, social and environmental levels. The findings from this qualitative study have directly informed the NIDUS intervention content, including modules on managing risk, communication with services, a local resource directory and getting out and about. They have also informed the training for facilitators on managing multiple and differing perspective and regular discussion on who attends session and how to meaningfully include the person living with dementia. We included vignettes and direct quotations from these interviews in the manuals to inform discussion and highlight the tensions and dilemmas that people may be experiencing, as well as potential solutions. The qualitative work reported in this paper also informed our overall approach in designing the NIDUS intervention: building on existing skills and resources and connecting with wider support networks.

### Strengths and limitations

We purposively sampled a diverse range of people living with dementia, family carers and health and social care professionals, accessing a breadth of viewpoints and perspectives contributing to the richness and relevance of the analysis [33]. We included the perspective of people living with dementia, often overlooked in research [34]. This relatively large qualitative dataset and involvement of a broad team encompassing academic, clinical and personal experience in the analysis adds credibility and validity to our findings. However, the study may have more resonance in the context of UK health and social care. Notably, nearly half of the family carers we interviewed came from South Asian backgrounds and we have explored how their cultural identities and values influenced their experiences, negotiation of the caring role and relationship with services elsewhere [35]. Additionally, ultimately we interviewed those who were keen and willing (and had the capacity to consent) to participate, therefore there is an inherent bias in our sample. It may be that those family carers and people living with dementia who chose to participate were less likely to be experiencing challenges or disagreements in relation to independence at home and it is likely that the health and social care professionals were particularly motivated or engaged in the topic. We did not directly observe how independence at home is enacted [33]. Triangulating with observations would have added to the richness of our accounts, and enabled us to include the perspective of people living with more advanced dementia who lacked capacity to consent.

## Conclusions

Our findings highlight the complexity involved in supporting and maintaining independence at home for people living with dementia. Psychosocial interventions must accommodate tensions between positive risk-taking and avoiding harm, facilitating autonomy and providing support. They should be adaptive and collaborative, combining self-management with flexible support and consider the emotional burden for family carers of supporting someone to live positively with risk.

## Supplementary information

**Additional file 1:** Semi-structured interview schedule.

**Additional file 2:** Checklist for Standards for Reporting Qualitative Research.

## Data Availability

The qualitative data used and analysed during the current study are available from the corresponding author on reasonable request.

## Abbreviations

FC: Family carer
HSCP: Health and Social Care Professional
NHS: National Health Service
NIDUS: New Interventions for Independence in Dementia
PLWD: Person Living with Dementia
