# Supplementary material for: “I just keep thinking that I don’t want to rely on people.” a qualitative study of how people living with dementia achieve and maintain independence at home: stakeholder perspectives
Source: BMC Geriatr. 2020 Jan 3;20:5. doi: 10.1186/s12877-019-1406-6 (PMC6942277; doi:10.1186/s12877-019-1406-6)
Supplement: Supplementary file 1 — Additional file 1: Semi-structured interview schedule. [file 12877_2019_1406_MOESM1_ESM.docx]

**Topic guides**

**Family carer interview topic guide**

**INTRODUCTION**

Thank you for agreeing to talk with me. This part of our study aims to help us understand better how to support people with dementia living in their own homes.

In order to make sure that I don’t miss anything, I will record our conversation on a digital recorder and then it will be professionally transcribed. Once the interview has been transcribed, I will ensure that everything will be anonymous so you can’t be identified. Once again, everything you tell me will be treated with complete confidence.

**INTERVIEW**

To start, I will ask some background information. Could you tell me a little about your relationship with [patient name]?

Do you live together?

How often do you see them?

What sort of support do they need?

Do you give them any support? What does this support look like?

Does anyone else support them e.g. other family members / paid carers?

- Prompt: If paid care how is this funded/arranged?

*e.g. self-funded – through a private agency or informal arrangement or*

*local authority (LA) funded – personal budget or arrangement with a specific provider by the LA*

Have you had any education/ support with understanding dementia?

-where from?

Has that been useful?

Does [patient name] have any other long term physical or mental health conditions (e.g. diabetes, hypertension, heart disease, asthma, COPD, other)?

What help do you and [name] have for their medical condition(s)?/ How do you and [name] manage it?

**Independence at home**

- To what extent is [the person you care for] currently able to live independently at home?
- What do you find independence means for the person you care for? What do you feel being independent looks like for them?
- Can you think of a time since [the person you care for] has had memory problems, when they have been able to achieve or do something independently that has been important for them? What happened? What made it easier?
- Can you think of a time since [the person you care for] has had memory problems, when they have not been able to achieve or do something independently that has been particularly difficult? What happened? What made it harder?
- What makes it harder / easier for the person you care for to live independently at home?
  - Prompt for: Impact of medical conditions/physical and mental health
  - Prompt for: professional home care

**Goals and priorities of people with dementia**

We are interested in what is most important to try to achieve when planning care for people living with dementia. Here are some of the areas that matter most to people:

- Moving around
- Cognition (memory and other areas of thinking)
- Being safe from falling
- Getting the right medical treatment for memory
- Getting the right medical treatment for other physical or mental health conditions
- Help at home
- Social activities
- Relationships
- Are there things you would add to this list? (prompt for behaviour, safety)
- What do you think is most important for [name of person with dementia]?
  - Why did you choose this?
- What else do you think is important for them? Why?
- Thinking more about the area selected, what do you think would be a realistic goal for [name] in the next 6 months?
  - What difference would this make to them (probe for independence/ safety/ social connection / health / being able to stay living at home)
  - What support might help [name] and you meet this goal?
  - Are there things that you think might make it easier or harder?
    - Prompt for:
    - Client factors: agitation (e.g. resisting care, verbal, physical aggression), personality/ culture/ethnicity/language, risks, physical or mental health
    - Family carer factors: (practical, emotional, relationship, health)
    - Home care agency/ management/legal/ NHS/ social services/ other
- [if home care recipient] How does the home care help [name] to work towards this? Are there ways of delivering it that would do this better?
- Are there any differences between what goals and priorities matter most to [the person you care for] and what matters to you as a carer?

**Interventions to help with meeting goals**

We want to develop a support programme to support people with dementia and their family carers to live independently at home and to meet the goals that they select.

What do you think this should include?

What do you think would work best?

What do you think you and the person you care for would find most useful?

We also want to develop some training for home carers working with clients living with dementia.

What do you think this should include?

Is there anything you wouldn’t want this to include or be like?

Thank you - Is there anything else you would like to add?

**Person living with dementia interview topic guide**

**INTRODUCTION**

Thank you for agreeing to talk with me. This part of our study aims to help us understand better how to support people with dementia living in their own homes.

In order to make sure that I don’t miss anything, I will record our conversation on a digital recorder and then it will be professionally transcribed. Once the interview has been transcribed, I will ensure that everything will be anonymous so you can’t be identified. Once again, everything you tell me will be treated with complete confidence.

**INTERVIEW**

**Independence at home**

We are interested in how you are able to live independently at home.

What do you do? Do you get any help from anyone else (paid carer / family member)? What do they do?

- Prompt: If paid care how is this funded/arranged?

*e.g. self-funded – through a private agency or informal arrangement or*

*local authority (LA) funded – personal budget or arrangement with a specific provider by the LA*

What can make it harder / easier to stay independent?

- - Prompt for: do you have any other long term physical or mental health conditions (e.g. diabetes, hypertension, heart disease, asthma, COPD, other)? Do these conditions make it harder / easier to stay independent?

**Your goals and priorities**

Here are some things that people with memory problems say are most important to them:

- Moving around
- Cognition (memory and other areas of thinking)
- Being safe from falling
- Getting the right medical treatment for memory
- Getting the right medical treatment for other physical or mental health conditions
- Help at home
- Social life and activities
- Relationships
- What is most important for you? (prompt relationships)
  - Why did you choose this?
  - What are you already doing (in relation to this goal) that is going well?
  - Is anything not going well?
  - How would you like things to change? What difference would this make to you (probe for independence/ safety/ being able to stay living at home)
  - Are there things that you think might make it easier or harder?
    - Prompt for: how person feels about their current situation, mental or physical health, culture/ethnicity/language, risks
    - How might your health affect you meeting the goal?/ What help do you have for your medical condition(s)?/ How do you manage it?
  - What support might help you do this?
  - [if home care recipient] Does the home care you receive help to work towards this? Are there ways it could be better?

**Interventions to help with meeting goals**

We want to develop a support programme to support people with dementia and their family carers to live independently at home and to meet the goals that they select.

What do you think this should include?

What would you find most helpful?

What would you enjoy?

Is there anything that you would not like to happen?

We also want to develop some training for home carers working with clients living with dementia.

What do you think this should include?

If you could, is there anything you would like to tell/teach/show home carers or care agency managers?

Thank you

Is there anything else you would like to add?

**Home manager/ health or social care professional interview topic guide**

**INTRODUCTION**

Thank you for agreeing to talk with me. This part of our study is to help us understand better how to support people living with dementia in their own homes.

In order to make sure that I don’t miss anything, I will record our conversation on a digital recorder and then it will be professionally transcribed. Once the interview has been transcribed, I will ensure that all identifying characteristics are removed. Once again, everything you tell me will be treated with complete confidence.

**Interview**

**To start, could you tell me a little about your work? How are you involved with caring for people living with dementia?**

*Probe for employment and social support*

- **How long have you been carrying out this role?**

**Independence at home**

- What do you find independence means for your clients with dementia? What do you feel being independent looks like for them?
- Can you think of a time when a client with dementia has been able to achieve or do something independently that has been particularly important for them? What happened?
- Can you think of a time when a client with dementia has been unable to achieve or do something independently that has been particularly difficult? What happened?
- What makes it harder / easier for your clients with dementia to live independently at home?

**Goals and priorities of people with dementia**

We are interested in what is most important to try to achieve when planning care for people living with dementia. Here are some of the areas that matter most to people with dementia:

- Moving around
- Cognition (memory and other areas of thinking)
- Being safe from falling
- Getting the right medical treatment for memory
- Getting the right medical treatment for other physical and mental health conditions
- Help at home
- Social activities
- Relationships
- Are there things you would add to this list? (prompt for behaviour, safety)
- Do you find there are sometimes differences between what goals and priorities matter most to the person with dementia and family carers? Can you say more about this?

**Interventions to help with meeting goals**

We want to develop a support programme to support people with dementia and their family carers to live independently at home and to meet the goals that they select.

What do you think this should include?

What do you think would work best?

We also want to develop some training for home carers working with clients living with dementia.

What do you think you and the person you care for would find most useful?

What do you think this should include?

Is there anything you wouldn’t want this to include or be like?

**Thank you**

**Is there anything else you would like to add?**
